# Evolution of Plant-Made Pharmaceuticals

**DOI:** 10.3390/ijms12053220

**Published:** 2011-05-17

**Authors:** David R. Thomas, Claire A. Penney, Amrita Majumder, Amanda M. Walmsley

**Affiliations:** School of Biological Sciences, Monash University, Clayton, Victoria 3800, Australia; E-Mails: david.r.thomas@monash.edu (D.R.T.); claire.penney@monash.edu (C.A.P.); amrita.majumder@monash.edu (A.M.)

**Keywords:** plant-made, recombinant protein, expression system

## Abstract

The science and policy of pharmaceuticals produced and/or delivered by plants has evolved over the past twenty-one years from a backyard remedy to regulated, purified products. After seemingly frozen at Phase I human clinical trials with six orally delivered plant-made vaccines not progressing past this stage over seven years, plant-made pharmaceuticals have made a breakthrough with several purified plant-based products advancing to Phase II trials and beyond. Though fraught with the usual difficulties of pharmaceutical development, pharmaceuticals made by plants have achieved pertinent milestones albeit slowly compared to other pharmaceutical production systems and are now at the cusp of reaching the consumer. Though the current economic climate begs for cautious investment as opposed to trail blazing, it is perhaps a good time to look to the future of plant-made pharmaceutical technology to assist in planning for future developments in order not to slow this technology’s momentum. To encourage continued progress, we highlight the advances made so far by this technology, particularly the change in paradigms, comparing developmental timelines, and summarizing the current status and future possibilities of plant-made pharmaceuticals.

## 1. Introduction

Vaccine development has advanced immensely over the last century. While conventional vaccines have reduced the burden of many life-threatening diseases, alternative methods for vaccine production have aimed to improve the number of diseases protected against, the quality and efficacy of the vaccines and their availability to a greater audience, especially to the less-privileged [1–3]. Several systems including *E. coli*, yeast, mammalian cells and insect cells, have been utilized for vaccine production. Each one has advantages and shortcomings with respect to recombinant protein production and a new vaccine candidate is often run through a battery of host production systems to determine the optimal one for expression.

Close to 21 years ago, transgenic plants were proposed as an alternative production system for pharmaceutical protein, with cited advantages of decreased cost, increased ease of delivery and scale up, and decreased risk of contamination with animal and human pathogens. We review the evolution of whole plants and plant cells as pharmaceutical production systems, highlighting shifting paradigms, comparing the timeline of development to competing production systems, and summarizing the current status and future possibilities of plant-made pharmaceuticals.

## 2. Development of the Plant-Made Vaccine Paradigm

**Figure 1.**
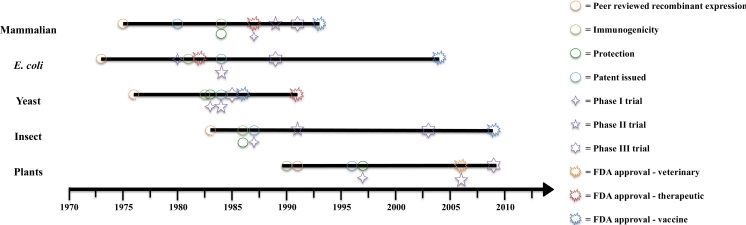
Comparison of the development of recombinant protein production systems.

In the early 1990s, three main groups were working to prove the concept of plant-made vaccines. The groups were all within academia and were under the tutelage of Charles Arntzen, Hilary Koprowski and Roy Curtiss. While the research of Dr. Arntzen’s group resulted in the first peer reviewed paper [4] Dr. Curtiss’s research resulted in the first patent [5] (Figure 1). At this early “edible vaccine” stage, it was proposed that antigen-producing fruit or vegetables would deliver vaccines in a safe and cost-effective manner, as well as increase the frequency of childhood vaccinations. In a competitive market with established production methods, the concept of using plants as an alternative vaccine source was not unrealistic and the prospect of using edible plant material to express and deliver vaccines was novel and appealing. It was proposed that the plants could be grown close to target populations using local farming techniques, and that this method would result in a cheap and easy mode of vaccination. It was thought that edible vaccines would reduce the cost of vaccination by cutting down on costs associated with transport, production, purification and other downstream processes used by conventional vaccines. Added to this was the advantage of eliciting mucosal immune response from the gastrointestinal tract, the site of entry of many pathogens. The basic concept of edible vaccines involved eating a piece of antigen containing fruit or vegetable to become vaccinated. However, this idea had several drawbacks, including difficulties in standardizing the vaccine dose and issues regarding the regulation of transgenic plants with added concerns of contaminating the food chain. This issue was highlighted by the “StarLink Affair” where non-genetically modified (GM) corn destined for human consumption was contaminated with a GM variety that had been approved for animal use but not human. Although the company was ordered to perform an extensive cleanup, the incident reflected poorly on the idea of food crops producing antigens [6].

Proposals soon followed that the transgenic plants producing pharmaceuticals should be grown in isolated from general food crops; and/or engineered to be male sterile [1,7]. Administration of vaccines supervised. Despite positive research outcomes, another limitation to using edible vaccines was soon greatly between different species and even from fruit to fruit and leaf to leaf within the same plant, making the realization of these shortcomings, the initial edible vaccine paradigm consisting of vaccine-delivering plant-derived vaccines involved the use of plants expressing a vaccine antigen; however instead of ingesting plant material was to be minimally processed, possibly through freeze drying, grinding and pooling to create to control dosage. This preparation could then be administered mixed in liquid that could be ingested under tablet [1]. Low-cost processing involving mild conditions to maintain the antigenicity of the expressed protein increased shelf life under a wider range of normal storage conditions [8].

A major hurdle for the oral delivery of any pharmaceutical is the digestive system degrading and digesting the protein before it can carry out its function. As a result, there can be significant variation in the quantity of antigen delivered to the immune system, necessitating administration of larger amounts of the vaccine formulation in order to offset the loss of antigens. The variability of responses between candidates, the large amount of antigen required, the concern regarding potentiating oral tolerance [9], and the continued concern of contamination of the food chain with pharmaceuticals led to the development of plant-based pharmaceuticals that are largely produced in non-food crops such as tobacco and are then highly purified for parenteral delivery. Presently there has been acceptance of recombinant, pharmaceutical proteins purified from plant sources for use in human clinical trials including Phase III [10–12], an extensive list of which is presented by Paul and Ma [13]. Dosage and quality control of these plant-made pharmaceuticals are controlled in a manner similar to their traditional counterparts.

## 3. Current Status of Plant-Made Pharmaceuticals

### 3.1. Plant-Made Vaccines

A search of the literature for plant-made antigens or vaccines results in multiple hits describing the expression of many different vaccine antigens in many different plant systems. It is not the ability of plant systems to express antigens that needs to be demonstrated to the wider protein production community, but rather the efficiency and efficacy of the resulting proteins. Further, the systems need to gain safety and regulatory approval, and demonstrate the capacity for economical production at large scales.

Originally, the major perceived disadvantage of the plant-made recombinant protein platform was its inability to compete with other recombinant protein production platforms with regards to the level of recombinant protein produced and the time taken to produce it. Compared to other platforms such as bacteria or animal cells, plants produced at least 10-fold less protein of interest, and it could take up to 18 months to produce a stably transformed plant line. However, this situation has become outdated with the refinement of transient plant transformation systems. Agroinfiltration can be used to deliver minimal, deconstructed plant virus-based expression vectors, such as the system based on a disabled cowpea mosaic virus RNA-2 by Sainsbury and colleagues [14] or the “magnifection” system developed by Icon Genetics [15,16], which has resulted in recombinant protein accumulating up to 80% of total soluble protein (TSP) in tobacco leaves [15].

D’Aoust *et al.* [17,18] made an important step with regards to utility of plant-based vaccines by demonstrating plants capable of acting as rapid response production systems. Protective antigens of multiple strains of influenza were transiently expressed in *Nicotiana benthamiana* using an *Agrobacterium*-mediated, transient expression system. The agro-infiltrated plants produced large amounts of protective antigen (50 mg/kg) from H5N1 (AIV) and H1N1 (human) strains. However the pertinent detail was that this was performed in less than three weeks from release of viral sequence to purified vaccine product. Thus this rapid vaccine production system would prove valuable in protecting against disease outbreaks and in enabling expedient mobilisation of personnel. The above mentioned plant-made influenza vaccine has completed Phase II human trials. Fraunhofer USA, Inc. has also used a transient plant transformation system to go from recently released H1 HA sequence to grams of purified protein in less than a month [19]. While these products would still have to acquire regulatory approval before use, this is also the case for current vaccines. Initial products would likely have to go through more rigorous trials, however once the safety of the platform is confirmed there is no reason that regulatory approval would take longer that for current technologies.

Recent advances in stable systems have resulted in much higher levels of recombinant protein accumulation. Utilization of sub-cellular targeting and chloroplast transformation has resulted in levels of up to 46% TSP being reported [20]. Stable expression of recombinant proteins such as vaccine antigens has also been actively pursued in seeds crops such as rice and maize, as these platforms produce high yields and are easy to scale up. Another benefit of seeds is their inherently stable nature. For example, a rice-based cholera vaccine, MucoRice-CTB, was shown to be stable at room temperature for 18 months, as well as being resistant to pepsin digestion [21]. This was later shown to be stable for up to three years, and gave protection to mice against both cholera and enterotoxigenic *Escherichia coli* (ETEC) [22]. Likewise, an ETEC subunit vaccine produced in soybean seeds was found to be stable over four years, as well as when formulated into soymilk [23]. The problems associated with seed systems are largely down to regulatory issues, as containment can be problematic with wind dispersal and seeds can remain viable in the soil after the crop has been harvested. Also, especially with crops such as rice and maize that are a staple food source for many, the potential for unwanted transgenes to enter the food supply could have major repercussions. However, these risks can be managed through the spatial and temporal segregation of crops as well as other physical and biological segregation techniques [24]. Chloroplast transformation can address some of these concerns regarding foreign gene transfer via pollen, due to the maternal inheritance of transgenes [25]. The potential for large-scale use of seed crops depends largely on the containment requirements decided on by regulatory agencies.

### 3.2. The Successful Plant-Made Vaccine

The world’s first licensing of an (injectable) plant-made vaccine occurred in early 2006 (Figure 1) [26]. On 31 January 2006, Dow AgroSciences LLC announced that it had received the world’s first regulatory approval for a plant-made vaccine from the United States Department of Agriculture. The developed plant-made vaccine combats Newcastle Disease Virus (NDV) and was made using a contained, plant-cell culture production system. A transgenic tobacco plant cell line that was grown as a suspension culture in a conventional bioreactor system was used to produce the plant-made NDV vaccine. Using this system, large quantities of vaccine can be produced in the bioreactor system in a relatively short time period of a few weeks. The resulting cells are harvested and minimally processed to provide a partially purified antigen for formulation into the final vaccine. Birds vaccinated subcutaneously with the plant-made vaccine were protected against lethal challenge to NDV [27]. While this was never brought forward to a commercially available product, the formulation was advanced through the USDA Center for Veterinary Biologics’ regulatory approval, demonstrating that plant-made vaccines could be developed within the existing regulatory framework.

### 3.3. Plant-Made Antibodies

As with plant-made vaccine antigens, there are many reports of plant-made antibodies in the literature, with applications ranging from diagnostics [28–31]; tumor targeting or cancer treatment [32–37]; prevention of tooth decay [38–40]; prevention of plant disease [41,42]; and preventing sexually transmitted disease [43–45] (see De Muynck *et al*. [46] for a comprehensive review). Different subclasses have been expressed (IgG, sIgA, IgM [46]) in different plant species however *Nicotiana* species (*N. tabacum* 58.1% or *N. benthamiana* 16.3%) dominate (74.4% total).

The refinement of transient, deconstructed plant viral transformation systems advanced the plant-made antibody field by allowing rapid and high-yield production of complex recombinant proteins such as antibodies. Initial attempts at the co-transformation of plant cells with multiple copies of the same virus vector containing different proteins (or different antibody fragments) resulted in the spatial separation of the different viral populations in the infiltrated tissues. However, Giritch and colleagues [47] solved this dilemma through using non-competing viral vectors such as tobacco mosaic virus and potato virus X.

Advanced plant and mammalian glycosylation differ in regards to the types of sugar moieties added and the types of linkages [48]. While this difference was not of consequence to the success of the plant-made antibodies described later, it is thought that this difference in glycosylation may result in the original problem with antibodies of non-human origin being seen as antigenic by the patient [49,50]. There is also some evidence that plant specific glycosylation motifs can elicit an immune response. However, plants have recently been genetically modified to mimic the typical animal glycosylation pattern thus preventing potential problems [46]. This was achieved by either inactivating native enzymes responsible for glycosylation [51,52], or by expressing heterologous enzymes responsible for mammalian-like glycosylation [53].

### 3.4. Successful Plant-Made Antibodies

Two plant-made antibody products have made it to human clinical trials. Planet Biotechnology Inc. produced the world’s first clinically tested antibody, CaroRx™ in tobacco. CaroRx™ specifically binds to the bacteria that cause tooth decay thereby preventing adhesion of the bacteria to teeth [54]. CaroRx™ is currently undergoing Phase II U.S. clinical trials under a U.S. Food and Drug Administration-approved Investigational New Drug application (Figure 1).

In July 2008 Large Scale Biology Corp. reported the success of the first human clinical trials testing a plant-made vaccine directed against cancer [55] (Figure 1). A transient, plant viral expression system produced patient-specific, recombinant, idiotype vaccines against follicular B cell lymphoma in tobacco. The vaccine was produced and purified within 12–16 weeks of receiving biopsy specimens before testing in a Phase I safety and immunogenicity clinical trial. Sixteen patients immunized with their own, individual therapeutic antigen showed no serious adverse effects yet 70% of patients developed cellular or humoral immune responses and 47% developed antigen-specific responses. Evidence was found that in this circumstance, immune responses were not directed toward plant-specific glycan structures (glycosylation) but rather to the idiotype sequence itself. In December 2009 Bayer started the clinical development of this plant-made antibody vaccine, successfully submitting a Phase I study protocol to the US FDA (United States Food and Drug Administration).

### 3.5. Additional Plant-Made Therapeutic Proteins

As is the case with antibodies, protein therapeutic use is limited by the shortfall in manufacturing capacity the high cost of production. Driven by our aging population, the 2003 market for therapeutic proteins grew by almost 19%, with predicted 2010 sales of over $90 billion [56]. The potential for future growth is dependent largely on the industry overcoming drug delivery challenges and cost issues.

As demonstrated by antibody production, plants excel at producing complex and “hard-to-make” therapeutic proteins. There have been many reports of therapeutic protein expression in plants, including anticoagulants [57]; thrombin inhibitors [57]; growth hormones [58]; blood substitutes [57,59]; collagen replacement [60]; antimicrobial agents [61]; and for treatment and/or prevention of neutropenia [62]; anemia [63]; hepatitis [57,64]; liver cirrhosis and burns [63]; cystic fibrosis, liver diseases and hemorrhage [62]; hypertension [62]; HIV [62]; diabetes [65]; Gaucher’s disease [11]; and organophosphate poisoning [66]. As with the previously described plant-made pharmaceuticals, the increase in recombinant protein produced due to the use of transient, deconstructed viral based expression systems has improved the likelihood of using plants as therapeutic protein production systems [67]. The low risks associated with some therapeutic proteins may also require less stringent containment, allowing seed crops to be grown on a large scale to produce large volumes of high-demand proteins.

### 3.6. Successful Plant-Made Therapeutics

The first report of a plant-made therapeutic (PMT) protein reaching Phase II human clinical trials was made by Biolex Therapeutics, Inc. regarding Locteron (Figure 1), a plant-made, controlled release, interferon alfa (IFN-α) treatment for chronic hepatitis C [64]. The current treatment of patients involves weekly administration of an IFN-α in combination with an antiviral drug. Unfortunately the antiviral drug (Ribavirin) is associated with significant side effects [68]. In 2005, 32 patients participated in Phase IIa clinical trials of Locteron. The IFN-α produced in the aquatic plant Lemna was administered fortnightly in combination with ribavirin in a randomised double blind study. An early virologic response was achieved by 100% (16/16) of hepatitis C patients treated with 480 and 640 μg doses. This is of importance, as early virologic responses have been established to be a pre-requisite for long-term response in hepatitis C patients. Phase IIb trials have since been performed with the results being released on March 31, 2011 at the 46th Annual Meeting of the European Association for the Study of the Liver (EASL) in Berlin [69].

The first plant-made therapeutic directed for human use to reach Phase III clinical trials was a carrot cell suspension derived, Gaucher’s disease therapeutic (Figure 1) developed by Protalix BioTherapeutics. The transgenic carrot cells expressed human glucocerebrosidase (human prGCD) and were grown as a cell suspension in a bioreactor system. The carrot cell suspension produced proteins that had consistent batch-to-batch enzymatic activity of a highly active product. The purified recombinant protein was tested in Phase I/II trials in 2006 [11] before entering Phase III trials in 2009 [70]. Thirty-one naive patients suffering from Gaucher disease were tested in a multi-centre, randomised, double blind trial. The primary endpoint (20% mean reduction from baseline in spleen volume) was achieved in prGCD treatment groups after only 6 months of therapy and furthermore the safety analysis showed that prGCD was well tolerated and no serious or severe adverse events were reported. Patients who successfully completed this study were granted expanded access some for over two and a half years. On December 1, 2009, Pfizer and Protalix entered into an agreement to develop and commercialise prGCD for the treatment of Gaucher’s disease. The agreement gives Pfizer exclusive worldwide licensing rights while Protalix retains commercialization rights in Israel. However, in early 2011 the FDA declined approval for the drug, asking for additional data from existing studies, but not requiring any additional trials.

SemBioSys have also completed a phase I-II trial of safflower-produced insulin, and found it to have a similar safety profile to current recombinant insulin [71]. The insulin is produced in oil bodies allowing for simplified extraction, and the plants have been grown in open fields. Also using seed crops, although this time in contained greenhouses, ORF Genetics produces various growth factors and cytokines in transgenic barley for use in cosmetics.

## 4. Comparing Development of Different Recombinant Production Systems

It can thus be seen that while plant-made vaccines have progressed at a relatively slow rate of development (Figure 1), the system has reached important milestones seen in the development of other pharmaceutical production systems. The most commonly used system for expression of recombinant protein is *Escherichia coli*. It was the first expression system introduced, with the pioneering work being undertaken by Stanley Cohen and colleagues in 1973, who demonstrated that a gene for ribosomal RNA from the South African clawed frog could be incorporated into and expressed by *E. coli* [72]. In terms of speed, bacterial cell cultures grow faster than insect cells, mammalian cells or yeast. However, they are unable to glycosylate proteins. For products where this is of importance, the problem can be overcome with the use of mammalian cells. Since their introduction in the mid-1970s [73], mammalian cell culture has become the second most used production system. This is despite inherent drawbacks to the system, including high fermentation costs, slow growth and the risk of viral infection. However, the history of non-recombinant mammalian cell cultures extends further back, with early developments in cell culture techniques driven by the need for an *in vitro* cell system for associated fields, such as virology and cell biology. For example, the first systematic review of cell culture conditions was in 1955 [74].

The main advantages of using yeast species such as *Saccharomyces cerevisiae* and *Pichia pastoris* for the production of recombinant pharmaceuticals are related to the fact that yeast is both a microorganism and a eukaryote. Yeast production systems have relatively high yields and low fermentation costs in comparison to mammalian systems, and as an additional benefit, and in contrast to *E. coli*, when yeast signal sequences are used, yeast species can secrete correctly folded and fully functional proteins into the medium, greatly decreasing the cost of purification [75]. However, for the production of glycoproteins, native high-mannose yeast glycosylation is not suitable for human use and represented a major limitation for yeast-based production systems before the development of glycoengineered yeast lines capable of producing humanised sialylated glycoproteins [76]. A relative newcomer to the protein production field is the baculovirus expression vector (BEV), a eukaryotic DNA viral vector that infects lepidopteran insect cells. While able to perform post-translational modifications of proteins, insect glycosylation is much simpler than that of mammalian cells.

The ultimate test of a production system is whether a product developed can be utilised in a clinical setting or as a veterinary product. As part of the transition from research laboratory to clinical trial, the product must receive the approval from pharmaceutical regulatory departments. There are three categories of products; diagnostic reagents, therapeutics and vaccines, each with its own set of conditions and regulations. The progress and applications of plant-derived pharmaceuticals as compared to insect, mammalian, *E. coli* and yeast is summarised in Figure 1. Cervarix, a bivalent vaccine against the human papillomavirus manufactured by GlaxoSmithKline, was the first vaccine made in insect cells to receive FDA approval in 2009. The use of insect cells as a production system for recombinant protein was first shown to be viable in 1983 [77], over 25 years before any recombinant protein pharmaceutical product received approval, and close to a decade before plant production was reported (Figure 1). It is worth noting that to date, no veterinary product produced in an insect production system has been granted regulatory approval.

The development of plant-made human and veterinary vaccines is an important step to concrete plant-based technology. The first human trials for a plant-derived vaccine were performed in 1997 [78]. Since that time, many products produced in plants have entered clinical trials and their efficacy tested.

The classes of drugs that face the greatest difficulty in receiving regulatory approval are human vaccines and therapeutics. The long, complicated and expensive process is designed to screen all potential new pharmaceuticals and to safe guard the public from drugs that have not been adequately researched. Any expression system used must be well characterized. As a result, many products do not reach clinical trial. The intricacy of the process and the costs involved often discourage smaller companies from investigating new pharmaceuticals. The production centers must meet strict standards for GMP, and Standard Operating Procedures must be developed and followed. While no plant-made human vaccine or therapeutic has as yet received regulatory approval, Protalix BioTherapeutics and Pfizer’s prGCD for the treatment of Gaucher’s disease has been granted Orphan drug designation and Fast Track status by the FDA. These achievements illustrate the “coming of age” of plant-based pharmaceutical manufacturing. Together with the approval of a veterinary vaccine, they illustrate that there is nothing about plant-based systems that poses insurmountable obstacles at the regulatory level. Bibliographic analysis has shown that after 20 years of active research, there has been no decline in publication activity related to plant-derived pharmaceuticals [10]. However it has been demonstrated many times that there is no universal recombinant production system that can guarantee high expression of a particular recombinant pharmaceutical, and as a result the choice of system will have to be approached on a case-by-case basis.

## 5. The Future of Plant Made Pharmaceuticals

The benefits of plant made pharmaceuticals have been pointed out repeatedly in the literature, with reviews on the technology and its advantages far outnumbering reviews on other systems. However, the technology is only now starting to be implemented due to investment by big pharmaceutical companies, such as Pfizer with prGCD, who have the funds and experience to deal with regulatory agencies needed to reach market approval. Once the infrastructure is in place for these ventures, it may become more common for plant systems to be the preferred method of protein production in the future. Now that plant made pharmaceuticals are approaching the market, it is perhaps a good time to look to the future of the technology. By looking at what can be achieved now and planning for future developments, the momentum that has slowly been building up to this point can be continued into new areas.

Plant-based systems have shown themselves able to reproduce a wide variety of human proteins. Beyond basic single peptide production, proteins that consist of multiple proteins have been produced and assembled in plants [38] as well as proteins requiring the co-expression of additional modifying enzymes [79], and indeed, entire synthesis pathways are now being transformed [80]. Unwanted posttranslational modifications can also be prevented by shutting down native modification pathways [51]. While retention of glycosylated protein in the endoplasmic reticulum has been used to prevent the plant specific glycosylation that occurs in the Golgi body, this method is not absolute, as some proteins pass through to the Golgi body before being recycled back to the endoplasmic reticulum (ER). This results in small, but detectable, quantities of protein with plant specific glycosylation [81]

The trend towards producing increasingly complex molecules is likely to continue, requiring both the expression of a precursor protein as well as the addition of modification pathways. Furthermore, target proteins may be modified by the addition of extra functional domains, altered sequences or optimized glycosylation to increase bioactivity or stability. For example, a human IgG produced in moss lacking plant specific *N*-glycosylation possessed a 40-fold increase on antibody-dependent cellular cytotoxicity compared to the mammalian version [82]. This is an area of great potential that is opening up as our understanding of molecular interactions increases.

While there has been a lot of work done on enhancing the transformation capabilities of plants, with many vectors being designed with various advantages and disadvantages [83], very little research has been done into optimizing the plants themselves. As plant bio-factories become more common, it seems an important next step is to optimize the plants used, and identify the conditions that suit the expression of specific proteins.

These optimizations can result from two mechanisms; by adjusting the conditions the plants are grown in or by genetically modifying the host plants. For example, there is a vast array of environmental factors that can affect the growth and metabolism of plants, and for the most part, these can be easily regulated [84]. However, there have been few studies on what the optimal growth conditions are for protein production, as opposed to plant growth. Certain promoters are more or less active under certain conditions, and the plant’s metabolism may vary greatly across environments [85]. Currently, whole plants are grown in conditions optimal for vegetative growth, although these conditions may not be ideal for transgene expression and accumulation. This may also differ between stable or transient production, as well as in response to tissue specific expression. Important factors may include photoperiod, light intensity, temperature, nutrient availability, humidity, carbon dioxide concentration and others, as well as interactions between them. Varying conditions may also be better suited to different stages of plant growth, from seedling to harvest. It is likely that expression levels could be boosted in many systems simply by adjusting these factors.

The second pathway for optimization is more likely to progress with the aid of larger biopharmaceutical companies. This entails the optimization of the plants themselves so that their characteristics are more amenable to producing recombinant proteins, such as modifying the metabolic pathways to produce human glycosylation patterns [53,86]. Harmful or disruptive native proteins can also be removed or down regulated. There is also a strong case for the addition of marker genes or DNA sequences to be included in transgenic plants, such as fluorescent proteins, which will allow the rapid and simple identification of recombinant plants in suspected escape cases [87]. Modifications can also be made to the life history of the plant, including delaying or preventing flowering, increasing leaf or fruit biomass, increasing growth rates, or adjusting the plants height. While terminator genes are frowned upon for agricultural GM plants, such a system would be ideal for recombinant protein production to prevent transgenes escaping into the wild.

The original idea of plant made vaccines was the ability to vaccinate someone by eating a piece of fruit or vegetable. However, recombinant plant proteins are currently only used after being highly purified. While raw edible vaccines are currently an unfeasible technology for human vaccines and therapeutics, it may not be necessary to fully isolate the target protein from plant material. A middle ground consisting of dried and ground plant material would be suitable for the oral delivery of vaccines and some therapeutics, and also allow for batch testing and analysis. In this case, a balance would have to be found between achieving the correct dose of vaccine while reducing the amount of any detrimental compounds. This could be greatly helped by optimizing the host plant’s characteristics with regard to the amount of detrimental metabolites it contains. Such oral vaccines could be produced cheaply and easily for use in developing nations [88]. This would also be an excellent option for the production of veterinary vaccines where recombinant feed could contain vaccine antigens. If yields can be better standardized, there is potential for the delivery of therapeutics in unprocessed plant material, especially for veterinary purposes or for products where the dosage has a wide active range. However, that would be not be a realistic option until wholly and partially purified products are on the market and shown to be safe and effective. It is likely that partially purified vaccines will first be introduced for veterinary purposes and then progress to humans once the technology gains acceptance. It is important that innovation continues in the field of plant-made pharmaceuticals and vaccines in order to confirm the technology’s potential to become a major platform for recombinant protein productions.
